# Bt, Not a Threat to *Propylea japonica*

**DOI:** 10.3389/fphys.2020.00758

**Published:** 2020-08-13

**Authors:** Chenchen Zhao, Linke Wu, Junyu Luo, Lin Niu, Chuanpeng Wang, Xiangzhen Zhu, Li Wang, Peng Zhao, Shuai Zhang, Jinjie Cui

**Affiliations:** ^1^State Key Laboratory of Cotton Biology, Institute of Cotton Research, Chinese Academy of Agricultural Sciences, Anyang, China; ^2^Hubei Insect Resources Utilization and Sustainable Pest Management Key Laboratory, College of Plant Science and Technology, Huazhong Agricultural University, Wuhan, China; ^3^Zhengzhou Research Base, State Key Laboratory of Cotton Biology, Zhengzhou University, Zhengzhou, China; ^4^College of Horticulture and Plant Protection, Yangzhou University, Yangzhou, China

**Keywords:** Bt proteins, bacterial diversity, detoxification genes, digestion genes, fitness

## Abstract

Given the ever-increasing commercial planting of transgenic plants across the world, an evaluation of their impacts on non-target organisms is as an important part of the risk assessment process. *Propylea japonica* is a dominant non-target predator and pollen feeder insect that is prevalent in Bt cotton fields, and it is thus in direct contact with Bt proteins. However, the effect of Bt proteins on *P. japonica* has not received much attention. In this study, the effects of Cry1Ac and/or Cry2Ab proteins on *P. japonica* were investigated from three aspects. First, no significant differences in the diversity of the microbiota nor change in species composition and community structure were observed among Cry protein treatments. Firmicutes are the most abundant bacterial phylum present in *P. japonica*, followed by Proteobacteria and Actinobacteria. The most abundant genus was *Staphylococcus*. Second, the expression levels of the detoxification and digestion-related genes did not change significantly in any Cry protein treatment. Third, none of the Cry proteins affected the population fitness of *P. japonica*. These results indicated that *P. japonica* was not sensitive to Bt proteins, suggesting that growing Bt cotton expressing Cry1Ac and/or Cry2Ab will pose negligible risks to *P. japonica*.

## Introduction

The global cultivation of genetically modified (GM) crops exceeded 191.7 million hectares in 2018 (the 23rd year of continuous biotech crop adoption), which substantially contributes to both increasing crop production and reducing poverty (ISAAA, 2018). This phenomenon has been especially true for major commercial crops like cotton (*Gossypium hirsutum* L.) and maize (*Zea mays* L.) where genetic engineering has contributed to both increased production and reduced reliance on insecticide treatments (Sanahuja et al., 2015). Transgenic plants comprise an increasing portion of cultivated crop plants grown worldwide, leading to both heightened concerns and increased scrutiny regarding their biosafety (Roberts, 1989; Shelton et al., 2002; Carriere et al., 2003; Lu et al., 2015). *Bacillus thuringiensis* (Bt) insect-resistant transgenic plants are highly popular and widely commercialized. Bt insect-resistant transgenic plants are highly popular and widely commercialized. Numerous studies have shown that Bt*-*transgenic cotton can suppress populations of a target pest with a narrow host range, resulting in increased yields (ISAAA, 2018) and great economic and ecological benefits (Wu et al., 2008).

*Bacillus thuringiensis* is a biopesticide that is considered a powerful tool to control pests in the order Lepidoptera, including butterflies, moths, and skippers, and is an environmentally friendly alternative to chemical pesticides (Wilson et al., 2013; Le Van et al., 2016). *Cry1Ac* and *cry2Ab* toxin genes have been used commercially to protect against Lepidopteran pest attack (Shelton et al., 2002), followed by Bt-transgenic insecticidal crop development from one gene as well as a fusion gene (Li et al., 2019). The impact of Bt-transgenic plants on non-target organisms is currently a major concern, and the effect of Bt on non-target predators is gaining increasing attention (Shelton et al., 2002). One key question is how much risk does a given level of Bt cotton pose on non-target insects that fulfill vital ecological functions? Evaluations of potential off-target effects of Bt proteins and GM organisms across different agricultural ecosystems are crucial (Li et al., 2017). Despite the fact that *Bt* cotton has been grown commercially for 20 years, data on Bt protein concentrations in herbivores and natural enemies are scarce, particularly for dual gene cotton (Meissle and Romeis, 2018). Thus, understanding whether Bt influences predators, and the extent to which this occurs, remains a major challenge for the development of robust and informative biosafety evaluation processes.

The ladybird *Propylea japonica* Thunberg (Coleoptera: Coccinellidae) is an indigenous natural enemy in China and a common and abundant species throughout East Asia that mainly preys on aphids, whiteflies, planthoppers, small caterpillars, and pollen in farmland ecosystems (Zhang L. et al., 2019). It functions as an indicator of potential adverse effects when GM crops are used in the field (Shelton et al., 2002). Moreover, the prey of *P. japonica* often consume Cry proteins present within Bt-transgenic cotton both indirectly (via prey, both larvae and adult are predaceous) and directly (via pollen consumption). Many studies have reported negligible phenotypic effects of Bt protein on *P. japonica* (Zhang et al., 2006; Li et al., 2015, 2017; Zhao et al., 2016). However, the molecular and bacterial responses of insects to Bt proteins have not been elucidated. Insects can protect themselves from secondary plant metabolites, toxic chemicals, and pathogens by regulating the expression of genes encoding detoxification and digestion responses (Bao et al., 2012). Cytochrome P450 monooxygenases (P450s), glutathione S-transferases (GSTs), carboxylesterase (CarE), and carboxyl/cholinesterase (CCE) have been reported to be involved in the xenobiotic metabolism of insects (Enayati et al., 2005; Ramsey et al., 2010; Gong et al., 2014; Gunderson et al., 2018). Despite a few examples, the effect of Bt protein on modifying the detoxification response in non-target insects has received scant attention (Mannakkara et al., 2013; Zhao et al., 2016).

Microbial symbionts are involved in most host life processes and play a crucial role in the growth and development, digestion, immunity, and other physiological functions of their hosts (Lemaitre and Hoffmann, 2007; Warnecke et al., 2007). In particular, some microbiomes have positively contributed to the detoxification of toxic proteins and compounds injected by insects (Douglas, 2015). Moreover, the gut microbiota of target insects appears to influence the host interaction with pathogenic microorganisms. Bt toxins can change the gut microbiota composition of Lepidopteran insects (Caccia et al., 2016), thereby altering insect susceptibility to Bt and its toxins due to gut microbiota changes (Broderick et al., 2004). The knowledge emerging from studies on symbiotic bacteria urges us to consider it as an important hidden role in Bt proteins and non-target insect interactions. Unfortunately, the effect of Bt proteins on bacterial diversity in non-target insects has rarely been manipulated experimentally, and such research may provide information about a novel aspect of non-target risk assessment of GM crops (Zhang S. et al., 2019).

To establish a baseline for experimental studies on the response of *P. japonica* to dietary intake of Bt toxins and demonstrate the effect of Bt toxins on the bacterial community for GM crop risk assessment, we systematically investigated the bacterial community to evaluate the effects of Cry1Ac and/or Cry2Ab toxins on initial instar larvae ladybird microbiota via 16S ribosomal RNA (16S rRNA) gene sequencing. In parallel, we characterized changes in the expression of key detoxification and digestion genes (e.g., *glutathione S-transferase* and *P450*) in *P. japonica* neonate fed with Bt proteins. We then measured the toxicity of Cry proteins on the population fitness (biological parameters, including developmental duration, survival rate, adult weight, fecundity, and oviposition period) of *P. japonica* across various life stages. This paper aimed to measure the effect of Bt proteins in different ways and provide technical support and experimental basis for the development of new methods for non-target risk assessment before transgenic crops expressing Bt proteins are approved for commercial cultivation.

## Materials and Methods

### Insect Rearing and Maintenance

*Propylea japonica* adults were originally collected from conventional cotton plants (CCRI49) cultivated at the Institute of Cotton Research, Chinese Academy of Agricultural Sciences (CAAS, 36°5′34.8′′ N, 114°31′47.19′′ E). The adults were bred in insect rearing cages (40 cm × 40 cm × 40 cm, 10 pairs per cage) and maintained on a diet of the pea aphid *Acyrthosiphon pisum.* Pea aphids were maintained on broad bean plants (*Vicia faba* L.) sowed in a greenhouse in the laboratory and grown under controlled conditions (25°C ± 1°C, 70% ± 10% RH, L:D = 14 h:10 h). After five generations, generation six (G6) *P. japonica* adults were used as the starting point for all experiments.

### Experimental Materials

Bt toxins, including Cry1Ac, Cry2Ab, and Cry1Ac + Cry2Ab proteins (high-purity, Supplementary Figure S1), were provided by the Institute of Plant Protection, CAAS. Bt proteins were solubilized in a solution of 2M sucrose (Álvarez-Alfageme et al., 2011) at a final concentration of 500 μg/mL, which was at least 10 times higher than that measured in Bt cotton pollen (Knight et al., 2013; Meissle and Romeis, 2018).

E-64 protease inhibitor (N-(*trans-*epoxysuccinyl)-L-leucine 4-guanidinobutylamide) was purchased from Sigma Aldrich (United States) and was diluted in 2M sucrose to a final concentration of 600 μg/ml. E-64 served as a positive control (Li et al., 2017).

### Preparation of Samples in Experiment

Eggs from the G6 *P. japonica* mix adults group were collected daily and kept in controlled conditions. Eggs were cleaned with 75% ethanol and then placed in sterile medium. The egg stage lasted for 1.6–2.2 days in the laboratory (observed data). Once larva emerged, neonates were transported immediately into individual centrifuge tubes (one neonate in one tube) and fed with 2M sucrose solution (which had the function of increasing food intake for *P. japonica*) containing (i) 500 μg/mL Cry1Ac; (ii) 500 μg/mL Cry2Ab; (iii) 500 μg/mL Cry1Ac + Cry2Ab (dual toxin); (iv) no added toxin (Control); and (v) 600 μg/mL E-64 protease inhibitor (positive control). About 2 μL of feeding solution was added to rearing containers daily.

After 2 days later, 40 larvae were collected from each treatment, immediately flash frozen in liquid nitrogen, and stored at −80°C until use (DNA and RNA extraction).

### Toxicity of Bt Proteins on Bacterial Community Diversity of *P. japonica*

#### DNA Extraction, PCR Amplification, and Sequencing

Total genomic DNA of four treatments (i, ii, iii, and iv with six repeats were used for each experiment) were extracted using the TIANamp Genomic DNA Kit (Tiangen Biotech Inc., China) according to the manufacturer’s instructions. Larval surfaces were cleaned with 75% ethanol and rinsed three times with sterile water before DNA extraction. An additional lysozyme (50 mg/mL) incubation step (30 min at 37°C) was included to break up Gram-positive bacterial cells. The quantity and quality of the DNA were measured with a NanoDrop 2000C spectrophotometer (Thermo Scientific, United States). The V3-V4 region of the 16S rRNA gene was amplified using 338F/806R primers (338F: 5′- ACTCCTACGGGAGGCAGCAG-3′, 806R: 5′- GGACTACHVGGGTWTCTAAT-3′) (Xu et al., 2016). Amplicon generation of PCR products, quantification and qualification were conducted as previously reported (Zhao et al., 2019). Purified amplicons were pooled in an equimolar concentration and paired-end sequenced (2 × 250) on an Illumina MiSeq platform (Illumina, San Diego, CA, United States) according to the standard protocols by Novogene Bioinformatics Technology Co., Ltd. (Beijing, China). The raw reads were deposited into the NCBI Sequence Read Archive (SRA) database (Accession Number: PRJNA591375).

#### Bioinformatics

Paired-end reads were assigned to samples based on their unique barcode and truncated by cutting off the barcode and primer sequence. QIIME standard operation procedure was used to progress raw sequence (Caporaso et al., 2010). Partial 16S rRNA bacterial sequences were filtered using Mothur (Schloss et al., 2009) with the inclusion criteria of mean quality score ≥20 and length ≥250 bp. To assess the taxonomy of the entire microbial community in ladybird beetles, reads were clustered into operational taxonomic units (OTUs) with a 97% similarity cut-off. Sequence analysis was performed by Uparse (Edgar, 2013). For the OTU richness and community diversity analyses, rarefied OTU tables were generated to prevent possible heterogeneity among samples due to differing numbers of sequences and to calculate community richness (observed OTUs, Chao1, ACE, and PD whole tree), diversity indices (Shannon and Simpson), and sequencing depth (Good’s coverage).

A Venn diagram was completed using Venn Diagram plotter software^1^ to illustrate the number of unique and shared OTUs across all samples evaluated. Bray-Curtis distance matrices were visualized by performing principal coordinate analysis (PCoA). UniFrac analysis was performed to compare microbial diversity (Hamady et al., 2009). The UniFrac distance is based on taxonomic relatedness. The weighted UniFrac metric considers the abundance of taxa presented in the sample, whereas the unweighted UniFrac does not, and it is thus highly sensitive to the presence of rare taxa (Edwards et al., 2015). A phylogenetic tree was constructed to compare differences between bacterial communities (Werner et al., 2011).

To test for community compositional differences, we used analysis of variance (Adonis) from R’s vegan package (Oksanen et al., 2017). The Adonis test is less sensitive to dispersion effects and is a more robust alternative to either analysis of similarities (ANOSIM) or multi-response permutation procedures (Chan et al., 2016). Values were compared using one-way ANOVA with Tukey’s HSD (honestly significant difference) or, when data were not normally distributed (*P* < 0.05), the non-parametric Kruskal–Wallis test. Meanwhile, we used the Kruskal–Wallis test to detect the species with different richness among four groups based on the microbial communities. These analyses were carried out by Statistics Analysis System (SAS 9.4, SAS Institute Inc.).

### RT-qPCR Analysis of Genes Related to Detoxification and Digestion Responses

To evaluate changes in gene expression in response to metabolic detoxification, the genes involved in this process were selected in accordance with prior studies (Tang et al., 2014; Zhao et al., 2016). Furthermore, we sought to identify genes putatively involved in detoxification and metabolism in *P. japonica* by using the available transcriptome database (unpublished data), including detoxification (carboxylesterase, *CarE*; glutathione s-transferase, *GST*; *P450;* and carboxyl/cholinesterase, *CCE*) and digestion (aminopeptidase N, *APN*; carboxypeptidase, *CP*; and trypsin, *TPs*, Supplementary Table S1). *P. japonica* candidate genes were evaluated to determine if their expression levels change in response to Cry1Ac, Cry2Ab, and Cry1Ac + 2Ab proteins by RT-qPCR.

Total RNA of four treatments (three repeats were used for each experiment) was extracted using SV total RNA Isolation System (Promega, United States) according to the manufacturer’s protocol. Single-stranded cDNA was synthesized using 1 μg of RNA from various samples with a reverse transcription system (PrimerScript^TM^ RT Master Mix, Takara, China). RT-qPCR was performed with a Mastercycler^®^ ep realplex system (Eppendorf, Germany) using GoTaq^®^ qPCR Master Mix (Promega, United States) according to the manufacturer’s protocol. The PCR cycling conditions were as follows: 94°C for 2 min followed by 40 cycles of 94°C for 10 s and 60°C for 15 s. Each reaction was performed in triplicate on three independent biological replicates. β-actin was used as a reference gene (GenBank: KJ522777.1) (Tang et al., 2014; Zhao et al., 2016; Supplementary Table S1). Transcript levels were calculated by the comparative 2−ΔΔCT method (Livak and Schmittgen, 2001).

### Toxicity of Bt Proteins to *P. japonica* Across Life Stages

*Propylea japonica* larvae were hatched from eggs and reared on 2M sucrose solution containing (i) 500 μg/mL Cry1Ac; (ii) 500 μg/mL Cry2Ab; (iii) 500 μg/mL Cry1Ac + Cry2Ab (dual toxin); (iv) no added toxin (Control); and (v) 600 μg/mL E-64 protease inhibitor (positive control) as described. Twenty individual *P. japonica* larvae were tested for each treatment (each treatment repeat three times).

Larval development and mortality were recorded twice per day (8:00 AM and 8:00 PM), and emerging adults were sexed and weighed (fresh weight, within 12 h). Emerging adults were randomly paired (18 pairs for each treatment) and maintained in 10 mL centrifuge tubes. The fecundity was observed daily for 20 days (from first oviposition day in each treatment), and the adults were weighed (dry weight).

Life table data were analyzed according to the two-sex life table theory in the TWO-SEX-MS Chart program (Chi, 1988). We used the bootstrap technique (Mignani and Rosa, 1995) with 100,000 bootstraps to obtain stable estimates (variance and standard error) of developmental time, sex ratio, and other population parameters. The bootstrap test was used to assess differences among treatments by TWO-SEX-MS Chart program. Tukey’s HSD test was used to evaluate the differences among weight.

## Results

### Effects of Bt Toxin on Bacterial Community of *P. japonica*

#### Overview of *P. japonica* Microbiotas

**FIGURE 1:**
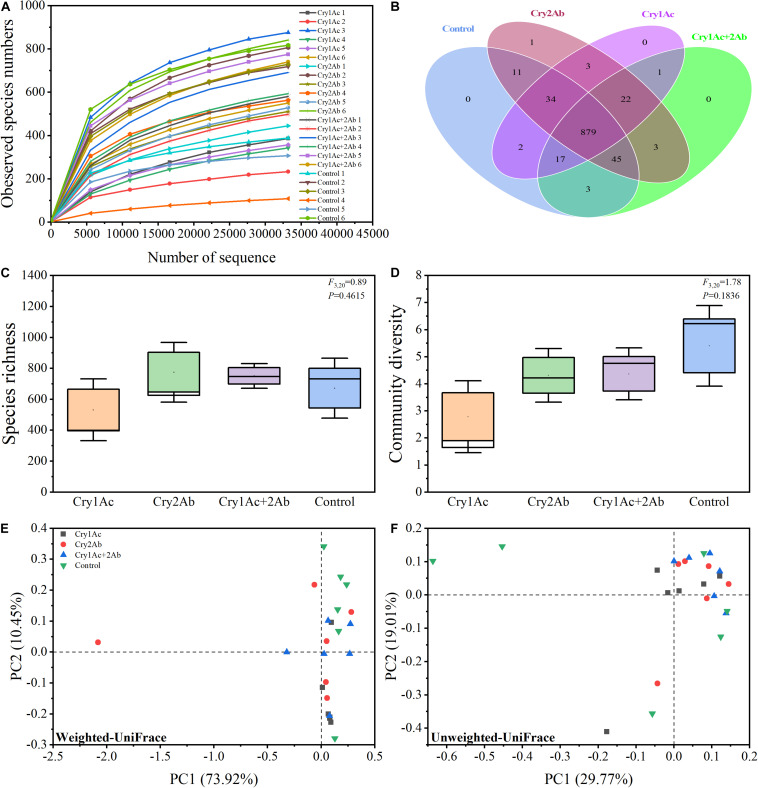
Bacterial diversity of *P. japonica* in response to consumption of Bt toxins. **(A)** Rarefaction curves based on species abundance data. Venn diagram **(B)** illustrating the number of bacteria shared and unique across four treatments. Boxplot of species richness (number of OTUs) **(C)** and community diversity measured by the Shannon index **(D)**. Different lowercase labels above each group indicate significant differences (one-way ANOVA, Tukey’s HSD test, *P* < 0.05) of group mean value. Principal coordinate analysis (PCoA) plot visualizing the data based on β-diversity metrics of weighted UniFrac **(E)** and unweighted UniFrac **(F)** distance. The percentage of variation explained by each axis refers to the fraction of the total variance of the data explained by the constrained factor.

We obtained a total of 2,293,040 valid sequence reads from the 24 samples evaluated. Six replicates were performed for each experimental treatment, and each individual sample comprised 40 ladybird larvae. On average, 81,624 effective sequence reads were achieved per sample, ranging from 44,798 reads to 93,780 reads with an average length of 253 bp (Supplementary Table S2). A total of 3,281 OTUs at 97% sequence similarity were identified. The rarefaction curves for all samples almost approached the saturation plateau, which indicate that 16S rRNA gene sequences were abundant and our analysis had adequate depth to capture most microbial diversity information (Figure 1A).

#### Effects of Cry1Ac, Cry2Ab and Dual Bt Toxins on the Microbiome of *P. japonica* Larvae

Our Venn diagram revealed only 0, 1, 0, and 0 OTUs unique to each of these four groups, respectively, with a total of 879 OTUs common to all groups (Figure 1B). Therefore, almost all species were shared in all four treatments.

Good’s coverage, which estimates the percentage of the total species represented in a sample, averaged 99.39%, thereby suggesting that the majority of microbial species present in *P. japonica* larvae were included in this study (Supplementary Table S2). Our data revealed that the microbiome remained largely static across the four treatments as observed by OTUs and Shannon’s diversity index. Larvae who fed on Bt proteins tended to harbor a relatively similar microbial diversity relative to controls (Figures 1C,D). The ACE, Chao1 estimator, Simpson’s diversity indices, and PD tree richness indices did not show any significant differences in bacterial community richness and diversity among the four treatments according to one-way ANOVA analysis (Supplementary Figure S2).

In the PCoA analyses based on the OTU (species) level, points that were close together represented samples that were highly similar in community composition. Compared with the control, no statistically significant differences were found in the bacterial community of *P. japonica* were associated with Cry1Ac, Cry2Ab, or dual Bt toxins (Figures 1E,F). ANOSIM and ADONIS analyses also indicated no statistically significant differences in the bacterial community in *P. japonica* between Bt treatment and control (*P* > 0.05, Table 1).

**TABLE 1:** Significance test of differences in bacterial community in *P. japonica* by ANOSIM and ADONIS.

	**ADONIS**					
**Sample group**	**ADONISM**			**Bray-Curtis**	**Weighted UniFrac**	**Unweighted UniFrac**
Cry2Ab vs. Control	R^2^	0.3667	R^2^	0.0939	0.3366	0.0910
	*P*	0.068	*P*	0.318	0.025	0.491
Cry2Ab vs. Cry1Ac + 2Ab	R^2^	0.1426	R^2^	0.1013	0.1256	0.1044
	*P*	0.056	*P*	0.245	0.092	0.408
Cry2Ab vs. Cry1Ac	R^2^	0.0889	R^2^	0.1195	0.1427	0.1313
	*P*	0.117	*P*	0.195	0.091	0.187
Control vs. Cry1Ac + 2Ab	R^2^	0.0278	R^2^	0.1164	0.1033	0.0898
	*P*	0.267	*P*	0.137	0.327	0.533
Control vs. Cry1Ac	R^2^	0.1722	R^2^	0.1459	0.1142	0.1112
	*P*	0.098	*P*	0.052	0.216	0.196
Cry1Ac + 2Ab vs. Cry1Ac	R^2^	0.0426	R^2^	0.0847	0.0805	0.0608
	*P*	0.247	*P*	0.563	0.539	0.881

Taken together, these results demonstrated that the bacterial community structures in *P. japonica* among the four treatments were not significantly different.

#### Comparing the Bacterial Community Diversity in *P. japonica* Larvae After Consuming Bt Toxins

**FIGURE 2:**
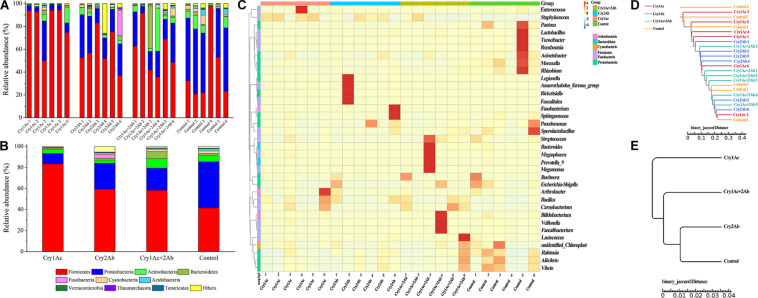
Bacterial composition of *P. japonica* in response to consumption of Bt toxins. Relative abundance of the dominant bacterial species found in *P. japonica* at the phylum level of each sample **(A)** and group **(B)**. Heatmap showing the relative abundance of dominant taxa at the genus level in each sample **(C)**. A dendrogram was prepared using the binary Jaccard distance to compare the similarity of the bacterial communities between samples **(D)** and groups **(E)**.

To visualize differences in the bacterial community, histograms of the top 10 bacterial genera were constructed among the different sample groups using the QIIME toolkit (Figures 2A,B). Taxonomic classification of the OTU representative sequences to the phylum level identified members of Firmicutes, Proteobacteria, Actinobacteria, Bacteroidetes, Cyanobacteria, and Fusobacteria. Firmicutes, Proteobacteria, and Actinobacteria were comprised in the core community (>87%) of *P. japonica* (Figure 1). When fed with Cry1Ac, the relative abundance of Firmicutes was 83.35% in *P. japonica* (Figure 2B), while the control group harbored 41.56% relative abundance of Firmicutes, ranking after Cry 2Ab (59.59%) and dual toxin (58.22%). By contrast, the abundance of Proteobacteria presented the following order (from highest to lowest): control group (43.85%), Cry2Ab (24.57%), dual toxin (21.12%), and Cry1Ac (10.07%). Actinobacteria observed in *P. japonica* showed different ranks in four treatments (in sequences): dual toxin (9.11%), control group (6.12%), Cry1Ac (3.67%), and Cry 2Ab (3.00%). However, Bacteroidetes was only represented by less than 1.53% of *P. japonica*, contrasting with the high value observed in the Cry1Ac group (6.70%). Despite the variation in abundance among treatments, the Wilcoxon signed-rank test showed no difference in phylum level between group (*P* > 0.05) (Supplementary Table S3).

In the genus level, *Staphylococcus*, *Bacillus* (Firmicutes), *Buchnera*, and *Acinetobacter* (Proteobacteria) were the predominant genera present in *P. japonica* (Supplementary Table S4). *Staphylococcus* was observed in all *P. japonica* with high abundance (56.46%, 39.07%, 19.24%, and 7.80%). Moreover, *Bacillus*, which was detected in the control group in abundances lower than 1%, was observed in greater abundance in the Bt group (7.56%, 6.92%, and 7.82%). Unlike other *P. japonica* fed with Bt protein, the bacterial composition in the control group showed greater evenness in the genus level. However, few genera were significantly associated with the Bt toxin treatment (Supplementary Table S5). *Carnobacterium* was more abundant in Cry1Ac and dual toxin group (12.50% and 16.55%, respectively), contrasting with the low values observed in both the Cry2Ab (0.95%) and control group (0.84%). *Moraxella* (Proteobacteria), which was detected in Bt-fed *P. japonica* larvae in abundances lower than 0.15%, was observed in greater abundances in the control group (0.99%). Others were observed with extremely lower relative abundance (e.g., *P. japonica* fed with dual toxin harbored *Microbacterium* with relative abundance of 0.01%, but it was 0.05% in the control group). The corresponding heatmap of the OTUs assigned to the genus level offered a detailed phylogenetic view of the bacterial community composition (Figure 2C).

Except for some samples that harbor special abundant genera, the microbiotas of four treatments were similar to one another. Samples failed to fall into clearly separate clades. The distance between samples indicates the differences between bacterial communities; however, a comparison of the phylogenetic distances between the groups (i.e., bacterial communities from each experimental group) indicated no clear separation of microhabitats among the four treatment groups (Figures 2D,E). They shared high similarity in their bacterial communities.

### Expression of Genes Associated With Detoxification and Digestion Responses

**FIGURE 3:**
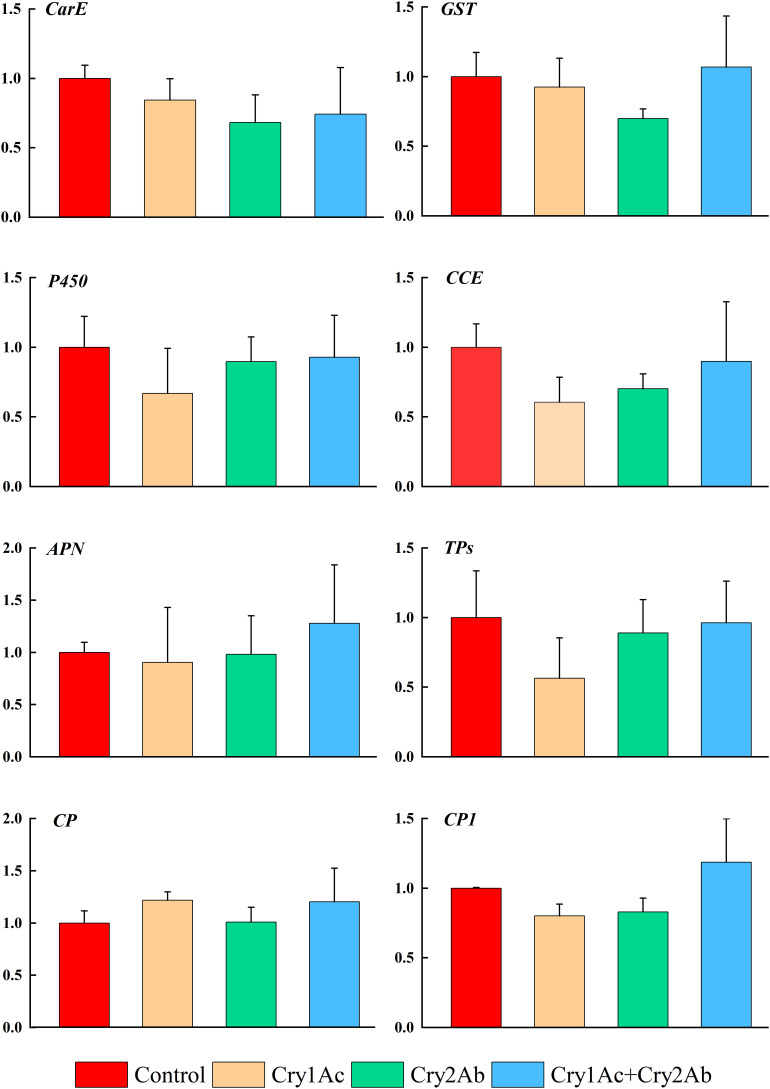
Relative expression levels of detoxification and digestion related genes of *P. japonica* when fed artificial diets containing Cry1Ac or/and Cry2Ab. The data represents as the mean ± SE. One-way ANOVA was performed, followed by Tukey HSD comparison test, *P* < 0.05.

In *P. japonica* fed artificial diets containing Cry1Ac, Cry2Ab, or Cry1Ac + Cry2Ab, genes that were associated with *P. japonica* detoxification responses (*CarE*, *GST*, *P450*, *CCE*) showed no significant difference compared with the control treatment (*P* > 0.05, Figure 3).

The expression levels of genes related to digestion responses (*APN*, *CP*, and *TPs*) also exhibited minimal changes between the Cry1Ac, Cry2Ab, and Cry1Ac + Cry2Ab treatments relative to the control; however, they did not exceed 1.3-fold (*P* > 0.05, Figure 3). RT-qPCR results indicated no significant differences among the eight genes involved in metabolism and detoxification in this study.

### Toxicity of Bt Proteins to *P. japonica* Across Life Stages

The larval and pupal development times did not significantly differ when *P. japonica* larvae were fed a sucrose solution-based diet that contained Cry1Ac or/and Cry2Ab protein (*P* > 0.05, Table 2). Meanwhile, the preadult survival, pupation, and eclosion rates did not significantly differ between each Cry protein treatment and the control (*P* > 0.05, Table 2). Moreover, no differences were found in adult fresh weight (HSD test; female: *F*_4_,_121_ = 1.05, *P* = 0.3830; male: *F*_4_,_88_ = 0.39, *P* = 0.8198) and dry weight (female: *F*_3_,_64_ = 0.56; *P* = 0.6454; male: *F*_3_,_63_ = 1.8, *P* = 0.1569) between each Cry protein treatment and the control. Remarkably, Cry protein could not affect the fecundity and oviposition days of *P. japonica* (*P* > 0.05).

**TABLE 2:** Biological parameters of *P. japonica* when fed a sucrose solution-based diet containing Cry1Ac and/or Cry2Ab or E-64.

**Treatment**		**Control**	**Cry1Ac**	**Cry2Ab**	**Cry1Ac ± 2Ab**	**E64**
Larval development duration (d)^a^	L1	1.77 ± 0.04(59)	1.79 ± 0.07(60)	1.73 ± 0.06(60)	1.80 ± 0.04(60)	1.98 ± 0.06(60)*
	L2	2.13 ± 0.06(59)	2.18 ± 0.07	2.11 ± 0.06(60)	2.06 ± 0.06(60)	2.50 ± 0.11(60)*
	L3	2.01 ± 0.05(59)	1.97 ± 0.05(58)	2.00 ± 0.06(58)	1.96 ± 0.04(60)	2.62 ± 0.12(60)*
	L4	3.59 ± 0.13(53)	3.57 ± 0.09(58)	3.42 ± 0.07(53)	3.63 ± 0.13(60)	4.28 ± 0.38(52)
**Pupal development duration (d) ^a^**		3.58 ± 0.08(53)	3.69 ± 0.07(58)	3.68 ± 0.06(53)	3.43 ± 0.08(56)	3.12 ± 0.25(34)
Rate (%) ^a^	Preadult survival	83.05 ± 4.88	85.01 ± 4.61	83.33 ± 4.83	81.66 ± 4.99	33.33 ± 6.07*
	Pupation (L1 to pupa)	89.83	96.67	88.33	93.33	56.67*
	Eclosion	92.45	87.93	94.34	87.50	58.82*
New adult fresh weight (mg)^b^	Female	4.16 ± 0.20(30)	4.24 ± 0.22(28)	4.36 ± 0.25(28)	4.35 ± 0.16(29)	3.62 ± 0.24(11)
	Male	3.85 ± 0.17(19)	3.91 ± 0.16(23)	3.95 ± 0.15(24)	3.66 ± 0.21(20)	3.81 ± 0.34(9)
Adult fresh weight (after oviposition for 20 days) (mg)^b^	Female	3.39 ± 0.22	3.44 ± 0.10	3.17 ± 0.12	3.32 ± 0.14	/
	Male	2.50 ± 0.11	2.63 ± 0.19	2.40 ± 0.09	2.84 ± 0.18	/
Fecundity ^a^		96.5 ± 15.21	91.1 ± 16.40	121.6 ± 12.35	94.2 ± 11.53	
Oviposition days^a^		10.4 ± 0.97	10.7 ± 1.21	12.0 ± 0.90	9.6 ± 0.9	

*Data are the mean ± SE. An asterisk denotes a significant difference between a toxin treatment to control. Sixty larvae were tested for each treatment. Sample of adult dry weight in E-64 treatment was insufficient for data analysis. a: Bootstrap test. b: HSD test. “(n)” represents the number of *P. japonica* at early stage.*

By contrast, *P. japonica* in the E-64 treatment exhibited a significantly increased larval development time (*P* < 0.05) and decreased pre-adult survival, pupation, and eclosion rates (*P* < 0.01) compared with the control (Table 2).

## Discussion

In our system, we focused on studying the effects of dietary consumption of Bt toxins on *P. japonica*, an important but non-target insect species, to better evaluate the environmental impacts of the commercial release of transgenic plants expressing Bt toxins in China. The concentrations of Bt toxins, namely, Cry1Ac, Cry2Ab, and Cry1Ac + Cry2Ab, used in our toxicity bioassays were at least 10 times higher than that measured in Bt cotton pollen, thus our study represents the worst-case scenario, increasing the certainty of environmental hazard assessments (Garcia-Alonso et al., 2006; Romeis et al., 2008). Using 16S rRNA sequencing, we evaluated both the structure and composition of the *P. japonica* bacterial community. Our results indicated that *P. japonica* bacterial community was not significantly altered in response to feeding insects diets rich in Bt toxins. We also extended this study to show that the expression of *P. japonica* gene levels involved in both metabolism and detoxification were not altered in response to the ingestion of Bt toxins. Meanwhile, our experimental results demonstrated that Cry protein did not affect *P. japonica* fitness.

Many studies have reported phenotypic effects on *P. japonica* of Bt protein, but the effect of Bt proteins on bacterial diversity in non-target insects has received scant attention. These diverse microbial communities provide important physiological functions for their insect hosts in many ways, including provision of nutritional supplements, enhancement of digestion, modulation of host immune homeostasis, protection from parasites and pathogens, insect mating and reproduction (Oliver et al., 2010; Cheng et al., 2017). Moreover, these bacterial communities are in a stable and controllable state when insects are reared indoors, which makes them suitable for assessing environmental risks of Bt protein (Zhang S. et al., 2019). These analyses revealed that enrichment of members of several bacterial phyla, including Firmicutes, Proteobacteria, Actinobacteria, Bacteroidetes, and Fusobacteria showed no difference. However, relative abundance of bacteria varied from food sources in genus level. Interestingly, *Carnobacterium* increased in abundance in *P. japonica* larvae fed Cry1Ac and dual toxin. Food has been reported to shape host bacterial communities (Priya et al., 2012; Christensen et al., 2019). Previous studies in *Hepialus gonggaensis* larvae demonstrated that most *Carnobacterium* species are harmless to their hosts (El Hassni et al., 2007), and their presence often results in a probiotic function and increased growth and improves the activity of intestinal digestive enzymes (Yin et al., 2011; Liang et al., 2019). *Staphylococcus* widely exists in mammals, insects, plants, and environmental samples; however, the role of *Staphylococcus* in insects remains unclear (Zhang S. et al., 2019). *Staphylococcus* was dominant in *P. japonica* and increased of proportion under feeding Bt toxin. *Moraxella*, *Staphylococcus*, and *Microbacterium* could have originated from ingested diet (Erban et al., 2016). A previous study suggested that Bt maize straw return causes changes in the bacterial community of *Eisenia fetida* casts, and differentiated bacterial species are associated with the mineralization, metabolic process, and degradation of plants residues (Shu et al., 2017). Thus, after ingesting toxic food, *P. japonica* may regulate the composition of their microbiota to enhance detoxification.

Insects have the ability to protect themselves from secondary plant metabolites, toxic chemicals, and pathogens by regulating the expression of genes encoding detoxification responses (Bao et al., 2012). To date, little is known about the detoxification and digestion response of non-target insects to Bt proteins. P450s, GSTs, and CCEs are major detoxifying enzyme families that are involved in the xenobiotic metabolism of insects (Feyereisen, 1999; Hayes et al., 2005; Hatfield et al., 2011; Van Leeuwen and Dermauw, 2016). CarE is involved in organophosphorus insecticide resistance and the metabolism of xenobiotic compounds in numerous insect species (Hatfield et al., 2011). APNs are widely studied not only because of their role in digestion but also for their involvement in the binding to Cry toxins (Bravo et al., 2007; Crava et al., 2010). CPs are involved in the degradation of dietary proteins (Bayes et al., 2005). TPs can mediate insect resistance to Bt toxins, allowing the insects to avoid exposure to high levels of toxin (Oppert et al., 1997). In the present study, we found that larvae that fed on Bt proteins failed to influence the expression of genes involved in detoxification and metabolism when compared with the larvae not exposed to Bt proteins. Similar results were noted for *CYP* of *Nilaparvata lugens* reared on transgenic rice (Mannakkara et al., 2013), *CarE* and *GST* of *P. japonica* fed on Cry1Ab toxin (Zhao et al., 2016). By contrast, the expression of *CYPs* was suppressed in *Helicoverpa armígera* (Muñoz et al., 2014). Our experimental results demonstrated that Cry1Ac, Cry2Ab, and dual proteins had no distinct effects on the detoxification and digestion-related gene regulation of *P. japonica*.

Many studies have reported ecological effects on non-target insects of Bt protein. In a previous study, Li and coauthors observed no adverse effects when *Coleomegilla maculata* larvae were fed Cry1Ac and Cry2Ab Bt toxins (Li et al., 2011). Furthermore, Cry1Ac toxin derived from *Bt* can be ingested and utilized as a supplementary dietary protein by *Plutella xylostella* (Sayyed et al., 2003). Zhang et al. (2006) showed that insects that consume Cry proteins via ingesting Bt-toxin Cry1Ac-treated pests in advanced larval stage exhibit no significant effect on their fitness. Similarly, Li et al. (2015, 2017) found evidence that consumption of Bt maize pollen containing Cry1Ie/Cry1C/Cry2A does not negatively affect *P. japonica*, and these levels of exposure (Cry1Ab/Cry1AcZM) do not change the survival rate, larval development period, adult weight, or spawning rates of these pollen feeders (Xie et al., 2018). Our results revealed no distinct differences in any of the *P. japonica* biological parameters between the Cry protein treatments and the control when directly fed a diet containing purified Cry1AC and/or Cry2A at concentrations that were >10-fold higher than those in pollen. Thus, Cry1Ac and/or Cry2Ab protein is unlikely to have detrimental effects on *P. japonica*.

Most previous studies have focused on the potential eco-toxicological effects of Bt crops on the life history traits or the population, while the effects of Bt protein on the *P. japonica* bacterial community have been rarely studied (Zhang S. et al., 2019). Food resources can cause shifts in bacterial communities of insects (Colman et al., 2012), and Bt toxins can change the gut microbiota composition of Lepidopteran insects (Caccia et al., 2016), which can strongly affect the susceptibility of the host to Bt protein in turn (Broderick et al., 2009). In this study, effects of Cry proteins on *P. japonica* were systematically investigated in terms of three aspects: bacterial community, expression detoxification and digestion-related genes, and population dynamics. The obtained results showed good agreement between all methods, thereby proving that the Cry protein had no adverse effect on *P. japonica*. Changeless population fitness, expression of detoxification and digestion-related genes in *P. japonica* may be determined by the unaltered symbionts. Insect symbionts are widely recognized as an important factor in host beneficial fitness (Pinto-Tomas et al., 2009; Sharon et al., 2010). Previous studies suggested that symbionts play a crucial role in host growth and development (Douglas, 2015). Moreover, the microbiome influences its host’s metabolism and detoxification (Shin et al., 2011; Kikuchi et al., 2012). The use of 16S rRNA high-throughput sequencing to evaluate the effect of Bt toxin on *P. japonica* is an easier, faster, and efficient method compared with other laboratory test methods. It will yield insight into new aspects on biosafety assessment.

## Conclusion

Our dietary exposure experiments highlight the dynamics of symbiosis and shed light on the change in biological parameters and genes responsible for digestion and detoxification of toxic proteins by *P. japonica.* These findings suggested that none of the biological parameters, expression levels of detoxification and digestion genes, and symbionts of *P. japonica* were affected by ingestion of Bt proteins. In particular, research on the bacterial community of *P. japonica* by emerging sequencing technology has provided evidence that 16S rRNA sequencing is a fast, reliable, and effective detection method to monitor effects on non-target species. This research established a foundation for the comprehensive understanding of the effects of Bt protein on *P. japonica* and provided a theoretical basis and novel aspect for the biosafety assessment of GM crops.

## Data Availability Statement

The datasets generated for this study can be found in the PRJNA591375.

## Author Contributions

LWu and CZ carried out the molecular lab work. CZ participated in the data analysis and drafted the manuscript. JL, LN, CW, XZ, LWa, and PZ carried out the statistical analyses. SZ and JC conceived of, designed, and coordinated the study, and helped draft the manuscript. All authors gave final approval for publication.

## Conflict of Interest

The authors declare that the research was conducted in the absence of any commercial or financial relationships that could be construed as a potential conflict of interest.

## Footnotes

1 https://omics.pnl.gov/software/venn-diagram-plotter
