# Supplementary material for: Bt, Not a Threat to Propylea japonica
Source: Front Physiol. 2020 Aug 13;11:758. doi: 10.3389/fphys.2020.00758 (PMC7438476; doi:10.3389/fphys.2020.00758)
Supplement: Supplementary file 1 [file Table_1.docx]

Supplementary Table 1. Primers used for qPCR analyses of detoxifying and digestive genes in *P. japonica*

| abbreviations | gene name | ID | Primer Sequence 5'–3' |
| --- | --- | --- | --- |
| β-actin | Beta-Actin | KJ522777.1 | 5’ GTTACTCTTTCACCACCACA3’ |
|  |  |  | 5’GGGCAACGGAATCTTT3’ |
| *GST* | Glutathione S-transferase | MK644177 | 5’ GGGTTATTAGTTTGAGGTTGAA3’ |
|  |  |  | 5’ACTCCACAGCAATCCATAA3’ |
| *CarE* | carboxylesterase | MK644178 | 5’ AAGACACTTGAGAAGGAGAA3’ |
|  |  |  | 5’ CAGATATAAGCAACGAGACAG3’ |
| *P450* | *Cytochrome P450* | MK644179 | 5’ TCTGTTGCGTTATGAGGT3’ |
|  |  |  | 5’ TTATGGTGAATATGTAAGCGATT 3’ |
| *CCE* | Carboxyl cholinesterase | MK644180 | 5’ AGCGAAGATTGCCTGTATA3’ |
|  |  |  | 5’ ATCCACCACCGAATATCC 3’ |
| *APN* | Aminopeptidase N | MK644181 | 5’ ATCGTCACAGGAACAAGT3’, |
|  |  |  | 5’ GGATGAGAACTGGACCTT 3’ |
| *CP* | *Carboxypeptidase* | MK644182 | 5’ GGATGAGAACTGGACCTT 3’ |
|  |  |  | 5’ TCTTCCACTCCTCCTCTAA 3’ |
| *CP1* | Carboxypeptidase | MK644182 | 5’ ACTATCTGTCCAGTAACGATT3’ |
|  |  |  | 5’ TCTTCCACTCCTCCTCTAA 3’ |
| *TPs* | Trypsin | MK644183 | 5’ AACTGTATCCTGTTATGTTGAC3’ |
|  |  |  | 5’ ACTTACGATGCTGTTCTGA 3’ |

Supplementary Table 2. General assembly statistics

| Sample Name | Raw data | Effective tags | OTU | Average length | Q20 | GC% | Effective% | God’s coverage |  |
| --- | --- | --- | --- | --- | --- | --- | --- | --- | --- |
| Cry1Ac 1 | 82,729 | 75,686 | 705 | 253 | 98.81 | 52.17 | 91.49 | 0.99793 |  |
| Cry1Ac 2 | 89,157 | 82,853 | 469 | 252 | 98.93 | 52.07 | 92.93 | 0.99905 |  |
| Cry1Ac 3 | 86,224 | 76,368 | 1,651 | 253 | 98.76 | 53.73 | 88.57 | 0.99681 |  |
| Cry1Ac 4 | 95,535 | 90,060 | 668 | 253 | 98.84 | 52.54 | 94.27 | 0.99831 |  |
| Cry1Ac 5 | 90,550 | 86,041 | 711 | 253 | 98.92 | 52.06 | 95.02 | 0.99850 |  |
| Cry1Ac 6 | 86,280 | 74,568 | 986 | 252 | 98.76 | 53.98 | 86.43 | 0.99757 |  |
| Cry2Ab 1 | 93,233 | 87,882 | 843 | 253 | 98.87 | 52.72 | 94.26 | 0.99798 |  |
| Cry2Ab 2 | 52,551 | 41,203 | 1,321 | 246 | 98.59 | 53.1 | 78.41 | 0.99845 |  |
| Cry2Ab 3 | 95,931 | 91,223 | 902 | 253 | 98.89 | 52.56 | 95.09 | 0.99779 |  |
| Cry2Ab 4 | 59,309 | 45,981 | 910 | 241 | 98.62 | 50.71 | 77.53 | 0.99798 |  |
| Cry2Ab 5 | 97,485 | 92,068 | 975 | 253 | 98.89 | 52.38 | 94.44 | 0.99725 |  |
| Cry2Ab 6 | 90,219 | 76,342 | 1,621 | 251 | 98.75 | 53.06 | 84.62 | 0.99586 |  |
| Cry1Ac+2Ab 1 | 82,388 | 73,947 | 989 | 253 | 98.81 | 52.28 | 89.75 | 0.99733 |  |
| Cry1Ac+2Ab 2 | 91,618 | 86,860 | 960 | 253 | 98.83 | 52.29 | 94.81 | 0.99815 |  |
| Cry1Ac+2Ab 3 | 98,400 | 82,609 | 1,257 | 253 | 98.78 | 52.78 | 83.95 | 0.99681 |  |
| Cry1Ac+2Ab 4 | 87,874 | 76,253 | 1,100 | 253 | 98.61 | 55.87 | 86.78 | 0.99708 |  |
| Cry1Ac+2Ab 5 | 78,131 | 65,475 | 1,235 | 248 | 98.66 | 53.49 | 83.8 | 0.99686 |  |
| Cry1Ac+2Ab 6 | 91,878 | 83,311 | 1,246 | 253 | 98.81 | 54.26 | 90.68 | 0.99725 |  |
| Control 1 | 92,344 | 86,789 | 742 | 253 | 98.74 | 52.26 | 93.98 | 0.99861 |  |
| Control 2 | 88,246 | 80,034 | 1,235 | 253 | 98.68 | 54.87 | 90.69 | 0.99782 |  |
| Control 3 | 86,252 | 78,434 | 1,311 | 252 | 98.67 | 54.34 | 90.94 | 0.99763 |  |
| Control 4 | 94,435 | 91,213 | 257 | 251 | 98.76 | 54.61 | 85.33 | 0.99913 |  |
| Control 5 | 74,394 | 68,861 | 788 | 254 | 98.52 | 52.84 | 85.77 | 0.99855 |  |
| Control 6 | 83,834 | 71,539 | 1,396 | 253 | 99.30 | 54.22 | 84.19 | 0.99610 |  |

Supplementary Table 3 Comparing the relative abundance of bacterial communities of *P. japonica* in phylum level

| Taxa | Mean (Relative abundance %) | | | | P | Q | P | Q | P | Q | P | Q | P | Q | P | Q |
| --- | --- | --- | --- | --- | --- | --- | --- | --- | --- | --- | --- | --- | --- | --- | --- | --- |
|  | Cry1Ac | Cry2Ab | Cry1Ac+2Ab | Control | Cry1Ac VS Control | | Cry2Ab VS Control | | Cry1Ac+2AbVS Control | | Cry1Ac VS Cry2Ab | | Cry1Ac VS Cry1Ac+2Ab | | Cry2Ab VS Cry1Ac+2Ab | |
| Firmicutes | 83.35% | 59.50% | 58.22% | 41.56% | 0.093 | 0.608 | 0.818 | 1 | 0.18 | 1 | 0.093 | 0.259 | 0.026 | 0.133 | 0.818 | 1 |
| Proteobacteria | 10.07% | 24.57% | 21.12% | 43.85% | 0.065 | 0.608 | 0.589 | 1 | 0.065 | 1 | 0.041 | 0.226 | 0.093 | 0.177 | 0.589 | 1 |
| Actinobacteria | 3.67% | 3.00% | 9.11% | 6.12% | 0.173 | 0.608 | 0.589 | 1 | 0.575 | 1 | 0.31 | 0.36 | 0.24 | 0.186 | 0.589 | 1 |
| Cyanobacteria | 0.88% | 1.07% | 1.88% | 2.59% | 0.485 | 0.746 | 0.937 | 1 | 0.818 | 1 | 0.589 | 0.41 | 0.31 | 0.186 | 0.937 | 1 |
| Bacteroidetes | 0.69% | 1.51% | 6.70% | 1.52% | 0.128 | 0.608 | 0.699 | 1 | 0.699 | 1 | 0.378 | 0.376 | 0.297 | 0.186 | 0.699 | 1 |
| Acidobacteria | 0.41% | 0.74% | 1.00% | 2.07% | 0.485 | 0.746 | 0.699 | 1 | 0.699 | 1 | 0.065 | 0.226 | 0.041 | 0.133 | 0.699 | 1 |
| Verrucomicrobia | 0.16% | 0.08% | 0.20% | 0.39% | 0.699 | 0.857 | 0.297 | 1 | 0.937 | 1 | 0.173 | 0.28 | 0.132 | 0.177 | 0.297 | 1 |
| Fusobacteria | 0.08% | 3.72% | 0.08% | 0.07% | 0.485 | 0.746 | 0.81 | 1 | 0.937 | 1 | 0.573 | 0.41 | 0.262 | 0.186 | 0.81 | 1 |
| Chloroflexi | 0.05% | 0.11% | 0.08% | 0.29% | 0.336 | 0.746 | 0.81 | 1 | 0.688 | 1 | 0.173 | 0.28 | 0.128 | 0.177 | 0.81 | 1 |
| Euryarchaeota | 0.04% | 0.02% | 0.01% | 0.01% | 1 | 0.912 | 0.389 | 1 | 0.341 | 1 | 0.864 | 0.463 | 0.34 | 0.186 | 0.389 | 1 |
| Gemmatimonadetes | 0.04% | 0.11% | 0.13% | 0.33% | 0.518 | 0.751 | 0.818 | 1 | 0.818 | 1 | 0.064 | 0.226 | 0.03 | 0.133 | 0.818 | 1 |
| Deinococcus-Thermus | 0.03% | 0.07% | 0.06% | 0.06% | 0.262 | 0.716 | 1 | 1 | 1 | 1 | 0.336 | 0.36 | 0.296 | 0.186 | 1 | 1 |
| Nitrospirae | 0.03% | 0.04% | 0.04% | 0.17% | 0.148 | 0.608 | 0.521 | 1 | 0.31 | 1 | 0.809 | 0.463 | 0.297 | 0.186 | 0.521 | 1 |
| Planctomycetes | 0.03% | 0.01% | 0.04% | 0.04% | 0.807 | 0.857 | 0.687 | 1 | 1 | 1 | 0.571 | 0.41 | 0.52 | 0.273 | 0.687 | 1 |
| Elusimicrobia | 0.02% | 0.04% | 0.07% | 0.16% | 0.871 | 0.857 | 0.748 | 1 | 0.809 | 1 | 0.229 | 0.319 | 0.149 | 0.18 | 0.748 | 1 |
| Chlamydiae | 0.01% | 0.01% | 0.02% | 0.02% | 0.732 | 0.857 | 1 | 1 | 0.685 | 1 | 0.324 | 0.36 | 0.324 | 0.186 | 1 | 1 |
| Tenericutes | 0.01% | 0.05% | 0.20% | 0.03% | 0.221 | 0.679 | 0.47 | 1 | 0.228 | 1 | 0.127 | 0.28 | 0.044 | 0.133 | 0.47 | 1 |
| Thermomicrobia | 0.01% | 0.06% | 0.01% | 0.01% | 0.864 | 0.857 | 0.744 | 1 | 0.616 | 1 | 0.622 | 0.413 | 0.682 | 0.343 | 0.744 | 1 |
| Armatimonadetes | 0.00% | 0.00% | 0.00% | 0.01% | 0.653 | 0.857 | 0.93 | 1 | 0.584 | 1 | 0.588 | 0.41 | 0.285 | 0.186 | 0.93 | 1 |
| Chlorobi | 0.00% | 0.00% | 0.00% | 0.03% | 0.34 | 0.746 | 0.256 | 1 | 0.727 | 1 | 0.445 | 0.41 | 0.116 | 0.177 | 0.256 | 1 |
| Deferribacteres | 0.00% | 0.00% | 0.01% | 0.00% | 0.087 | 0.608 | 0.157 | 1 | 0.22 | 1 | 0.181 | 0.28 | 0.06 | 0.145 | 0.157 | 1 |
| JL-ETNP-Z39 | 0.00% | 0.00% | 0.03% | 0.01% | 0.73 | 0.857 | 0.266 | 1 | 0.678 | 1 | 0.787 | 0.463 | 0.316 | 0.186 | 0.266 | 1 |
| Saccharibacteria | 0.00% | 0.00% | 0.01% | 0.10% | 0.367 | 0.746 | 1 | 1 | 0.326 | 1 | 1 | 0.516 | 1 | 0.447 | 1 | 1 |
| Synergistetes | 0.00% | 0.03% | 0.00% | 0.00% | 0.859 | 0.857 | 0.214 | 1 | 0.248 | 1 | 0.663 | 0.42 | 0.282 | 0.186 | 0.214 | 1 |
| Thaumarchaeota | 0.00% | 0.38% | 0.02% | 0.07% | 0.171 | 0.608 | 0.126 | 1 | 0.624 | 1 | 0.025 | 0.226 | 0.304 | 0.186 | 0.126 | 1 |
| WD272 | 0.00% | 0.00% | 0.00% | 0.02% | 0.441 | 0.746 | 0.787 | 1 | 0.753 | 1 | 0.859 | 0.463 | 0.855 | 0.397 | 0.787 | 1 |
| Other | 0.41% | 4.86% | 0.96% | 0.47% | 0.936 | 0.887 | 1 | 1 | 0.575 | 1 | 0.589 | 0.41 | 0.818 | 0.395 | 1 | 1 |

Supplementary Table 4 Relative abundance of bacterial communities of *P. japonica* in genus level of top 35

| Taxonomy | Cry1Ac | Cry2Ab | Cry1Ac+2Ab | Control |
| --- | --- | --- | --- | --- |
| *Staphylococcus* | 56.46% | 39.07% | 19.24% | 7.80% |
| *Acinetobacter* | 1.15% | 1.87% | 1.47% | 5.83% |
| *Escherichia-Shigella* | 0.95% | 3.02% | 3.51% | 5.73% |
| *Buchnera* | 0.43% | 2.04% | 3.73% | 4.31% |
| *Lactobacillus* | 0.23% | 0.56% | 0.69% | 4.25% |
| *Pseudomonas* | 0.73% | 2.77% | 0.84% | 2.57% |
| *Vibrio* | 0.57% | 1.11% | 1.33% | 2.36% |
| *Romboutsia* | 0.09% | 0.05% | 0.11% | 1.98% |
| *Bacillus* | 7.56% | 6.92% | 7.82% | 1.96% |
| *Bifidobacterium* | 0.56% | 0.25% | 6.51% | 1.94% |
| unidentified_Chloroplast | 0.69% | 0.79% | 1.32% | 1.84% |
| *Pantoea* | 0.08% | 0.11% | 0.17% | 1.83% |
| *Ralstonia* | 0.58% | 1.09% | 1.22% | 1.78% |
| *Sporolactobacillus* | 0.36% | 0.58% | 0.65% | 1.65% |
| *Aliivibrio* | 0.28% | 0.56% | 0.81% | 1.34% |
| *Turicibacter* | 0.03% | 0.03% | 0.04% | 1.04% |
| *Rhizobium* | 0.09% | 0.17% | 0.15% | 1.01% |
| *Moraxella* | 0.13% | 0.14% | 0.12% | 0.99% |
| *Enterococcus* | 3.33% | 0.28% | 0.31% | 0.97% |
| *Streptococcus* | 0.44% | 0.17% | 1.49% | 0.84% |
| *Carnobacterium* | 12.50% | 0.95% | 16.55% | 0.84% |
| *Arthrobacter* | 1.28% | 0.50% | 0.27% | 0.53% |
| *Bacteroides* | 0.18% | 0.10% | 3.07% | 0.52% |
| *Sphingomonas* | 0.22% | 1.27% | 0.26% | 0.50% |
| *Veillonella* | 0.27% | 0.10% | 1.62% | 0.22% |
| *Lactococcus* | 0.05% | 0.13% | 0.89% | 0.17% |
| *Faecalibacterium* | 0.05% | 0.11% | 0.82% | 0.12% |
| *Megasphaera* | 0.12% | 0.06% | 1.15% | 0.09% |
| *Legionella* | 0.02% | 0.69% | 0.06% | 0.08% |
| *Megamonas* | 0.07% | 0.07% | 2.28% | 0.08% |
| *Prevotella_9* | 0.10% | 0.03% | 3.19% | 0.07% |
| *Fusobacterium* | 0.03% | 3.69% | 0.06% | 0.05% |
| *Rickettsiella* | 0.03% | 2.51% | 0.02% | 0.03% |
| *Anaerorhabdus_furcosa_group* | 0.02% | 0.71% | 0.01% | 0.01% |
| *Faecalitalea* | 0.01% | 0.55% | 0.01% | 0.01% |
| Others | 10.31% | 26.93% | 18.21% | 44.68% |

Supplementary Table 5 Bacterial communities with significantly different of *P. japonica* in genus level

| phylum | genus | Mean (Relative abundance %) | Mean (Relative abundance %) | P | Q |
| --- | --- | --- | --- | --- | --- |
|  |  | Cry1Ac+2Ab | Control |  |  |
| Firmicutes | *Carnobacterium* | 16.55% | 0.84% | 0.013 | 1 |
| Proteobacteria | *Moraxella* | 0.12% | 0.99% | 0.016 | 1 |
| Bacteroidetes | *Owenweeksia* | 0.02% | 0.00% | 0.037 | 1 |
| Actinobacteria | *Microbacterium* | 0.01% | 0.05% | 0.041 | 1 |
| Actinobacteria | *Corynebacterium1* | 0.19% | 0.73% | 0.041 | 1 |
|  |  | Cry2Ab | Control |  |  |
| Firmicutes | *Coprobacillus* | 0.04% | 0.00% | 0.025 | 1 |
| Proteobacteria | *Moraxella* | 0.14% | 0.99% | 0.041 | 1 |
|  |  | Cry1Ac | Control |  |  |
| Firmicutes | *Carnobacterium* | 12.50% | 0.84% | 0.020 | 1 |
| Bacteroidetes | *Parabacteroides* | 0.01% | 0.11% | 0.024 | 1 |
| Deferribacteres | *Mucispirillum* | 0.00% | 0.00% | 0.025 | 1 |
| Proteobacteria | *Moraxella* | 0.13% | 0.99% | 0.026 | 1 |
| Firmicutes | *Ruminococcus1* | 0.00% | 0.01% | 0.028 | 1 |
| Proteobacteria | *Stenotrophomonas* | 0.14% | 1.11% | 0.037 | 1 |
| Proteobacteria | *Shinella* | 0.00% | 0.04% | 0.039 | 1 |
|  |  | Cry2Ab | Cry1Ac+2Ab |  |  |
| Firmicutes | *Carnobacterium* | 0.95% | 16.55% | 0.004 | 1 |
| Firmicutes | *RuminococcaceaeUCG-010* | 0.00% | 0.02% | 0.020 | 1 |
| Proteobacteria | *Paracocccus* | 0.12% | 0.05% | 0.026 | 1 |
| Bacteroidetes | *Tunicatimonas* | 0.02% | 0.00% | 0.027 | 1 |
| Firmicutes | *Oxalophagus* | 0.01% | 0.00% | 0.028 | 1 |
| Firmicutes | *Clostridiumsensustricto1* | 0.03% | 0.10% | 0.029 | 1 |
| Proteobacteria | *Dyella* | 0.02% | 0.07% | 0.030 | 1 |
| Firmicutes | *Clostridiumsensustricto12* | 0.02% | 0.00% | 0.036 | 1 |
| Firmicutes | *Gemella* | 0.00% | 0.02% | 0.040 | 1 |
|  |  | Cry1Ac | Cry1Ac+2Ab |  |  |
| Proteobacteria | *Dyella* | 0.01% | 0.07% | 0.019 | 0.630 |
| Firmicutes | *Pseudobutyrivibrio* | 0.01% | 0.06% | 0.019 | 0.630 |
| Firmicutes | *Kurthia* | 0.00% | 0.02% | 0.024 | 0.630 |
| Actinobacteria | *Kineosporia* | 0.00% | 0.00% | 0.025 | 0.630 |
| Tenericutes | *Ureaplasma* | 0.00% | 0.02% | 0.028 | 0.630 |
| Actinobacteria | *Acidothermus* | 0.04% | 0.13% | 0.030 | 0.630 |
| Firmicutes | *Anaerostipes* | 0.00% | 0.04% | 0.040 | 0.630 |
| Firmicutes | *Lactococcus* | 0.05% | 0.89% | 0.041 | 0.630 |
| Firmicutes | *Blautia* | 0.09% | 0.71% | 0.041 | 0.630 |
| Firmicutes | *Peptoclostridium* | 0.01% | 0.05% | 0.043 | 0.630 |
| Acidobacteria | *Acidobacterium* | 0.01% | 0.02% | 0.043 | 0.630 |
| Firmicutes | *Dorea* | 0.01% | 0.05% | 0.043 | 0.630 |
| Proteobacteria | *Sorangium* | 0.02% | 0.05% | 0.045 | 0.630 |
| Firmicutes | *RuminococcaceaeUCG-010* | 0.00% | 0.02% | 0.045 | 0.630 |
|  |  | Cry1Ac | Cry2Ab |  |  |
| Firmicutes | *Carnobacterium* | 12.50% | 0.95% | 0.009 | 0.692 |
| Proteobacteria | *Defluviicoccus* | 0.00% | 0.01% | 0.009 | 0.692 |
| Actinobacteria | *Nakamurella* | 0.00% | 0.02% | 0.020 | 0.692 |
| Proteobacteria | *Massilia* | 0.12% | 0.34% | 0.026 | 0.692 |
| Proteobacteria | *Paracocccus* | 0.03% | 0.12% | 0.030 | 0.692 |
| Proteobacteria | *Cupriavidus* | 0.00% | 0.01% | 0.037 | 0.692 |
| Proteobacteria | *Ochrobactrum* | 0.00% | 0.02% | 0.040 | 0.692 |
| Proteobacteria | *Sphingomonas* | 0.22% | 1.27% | 0.041 | 0.692 |
| Proteobacteria | *Silanimonas* | 0.00% | 0.01% | 0.048 | 0.692 |
