# Supplementary figures and images for: Bt, Not a Threat to Propylea japonica
Source: Front Physiol. 2020 Aug 13;11:758. doi: 10.3389/fphys.2020.00758 (PMC7438476; doi:10.3389/fphys.2020.00758)

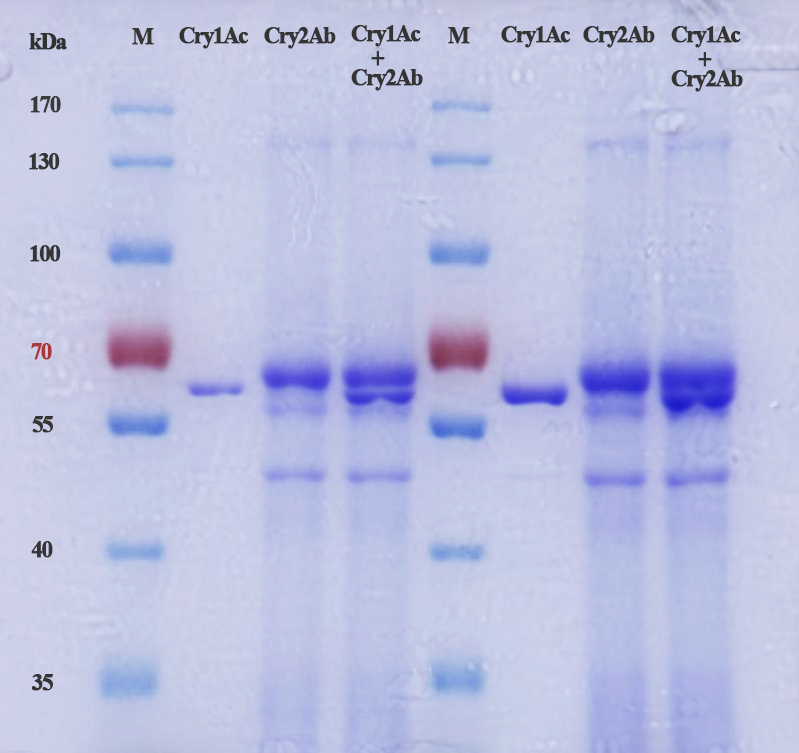

Supplement: Supplementary file 2 [file Image_1.JPEG]
